# Case Report: BAP1 Mutation and RAD21 Amplification as Predictive Biomarkers to PARP Inhibitor in Metastatic Intrahepatic Cholangiocarcinoma

**DOI:** 10.3389/fonc.2020.567289

**Published:** 2020-11-27

**Authors:** Francesco Sabbatino, Luigi Liguori, Umberto Malapelle, Francesca Schiavi, Vincenzo Tortora, Valeria Conti, Amelia Filippelli, Giampaolo Tortora, Cristina R. Ferrone, Stefano Pepe

**Affiliations:** ^1^ Department of Medicine, Surgery and Dentistry “Scuola Medica Salernitana”, University of Salerno, Salerno, Italy; ^2^ Oncology Unit, University Hospital San Giovanni di Dio e Ruggi D’Aragona, Salerno, Italy; ^3^ Department of Clinical Medicine and Surgery, University of Naples “Federico II”, Naples, Italy; ^4^ Public Health, University of Naples “Federico II”, Naples, Italy; ^5^ Familial Cancer Clinic and Oncoendocrinology, Veneto Institute of Oncology IOV–IRCCS, Padua, Italy; ^6^ Clinical Pharmacology and Pharmacogenetics Unit, University Hospital “San Giovanni di Dio e Ruggi D’Aragona”, Salerno, Italy; ^7^ Oncologia Unit, Fondazione Policlinico Universitario A. Gemelli IRCCS, Università Cattolica Del Sacro Cuore, Roma, Italy; ^8^ Department of Surgery, Massachusetts General Hospital, Harvard Medical School, Boston, MA, United States

**Keywords:** BAP1, precision oncology, cholangio carcinoma, Poly ADP ribose polymerase (PARP) inhibitor, RAD21, olaparib

## Abstract

**Introduction:**

Intrahepatic cholangiocarcinoma (ICC) is a rare hepatobiliary cancer characterized by a poor prognosis and a limited response to conventional therapies. Currently chemotherapy is the only therapeutic option for patients with Stage IV ICC. Due to the poor response rate, there is an urgent need to identify novel molecular targets to develop novel effective therapies. Precision oncology tests utilizing targeted next-generation sequencing (NGS) platforms have rapidly entered into clinical practice. Profiling the genome and transcriptome of cancer to identify potentially targetable oncogenic pathways may guide the clinical care of the patient.

**Case presentation:**

We present a 56-year-old male patient affected with metastatic ICC, whose cancer underwent several precision oncology tests by different NGS platforms. A novel BAP1 mutation (splice site c.581-17_585del22) and a RAD21 amplification were identified by a commercial available platform on a metastatic lesion. No germline BAP1 mutations were identified. Several lines of evidences indicate that PARP inhibitor administration might be an effective treatment in presence of BAP1 and/or RAD21 alterations since both BAP1 and RAD21 are involved in the DNA repair pathway, BAP1 interacts with BRCA1 and BRCA1-mediated DNA repair pathway alterations enhance the sensitivity to PARP inhibitor administration. In this case, after failing conventional therapies, patient was treated with PARP inhibitor olaparib. The patient had a partial response according to RECIST criteria with an overall survival of 37.2 months from the time of diagnosis of his ICC. Following 11.0 months on olaparib treatment, sustained stable disease control is ongoing. The patient is still being treated with olaparib and no significant toxicity has been reported.

**Conclusion:**

These findings have clinical relevance since we have shown PARP inhibitor as a potential treatment for ICC patients harboring BAP1 deletion and RAD21 amplification. We have also highlighted the utility of NGS platforms to identify targetable mutations within a cancer.

## Introduction

Cholangiocarcinoma (CCA) is historically classified by location into intrahepatic, perihilar (or Klatskintumor) and distal cancers. Intrahepatic cholangiocarcinoma (ICC) is the second most common primary intrahepatic tumor, with an estimated incidence of 1.6 per 100,000/year in the United States (1). Unfortunately, ICC carries an extremely poor prognosis with an overall 5-year survival of 5–15% (1). For patients with early stage ICC, surgical resection of the cancer and removal of local lymph nodes remains the only curative option (2). However, even with a complete resection, most patients succumb to both loco-regional and distant metastases (3). Unfortunately, most patients present with advanced disease. Palliative chemotherapy is of limited efficacy (4), highlighting the urgent need for novel effective therapies.

Different cancers express different oncogenic alterations which drive tumor progression. Several lines of evidences demonstrate that some of these alterations can be effectively targeted by tailored targeted agents, improving the overall survival of treated patients (5). These results have increased the use of precision oncology tests by targeted next-generation sequencing (NGS) platforms into clinical practice, to inform clinicians in making appropriate therapeutic decisions (6). Unselected ICC patients have been often included in “basket” trials (7), most of which have unfortunately failed to demonstrate a clinical benefit (7). As a result, there is a high interest to identifying oncogenic alterations in ICC to design potentially effective strategies in biomarker-enriched populations.

NGS of ICC has already allowed identification of molecular alterations which are involved in ICC carcinogenesis such as those in *KRAS*, *BRAF*, *IDH1, IDH2*, *EGFR*, *FGFR2*, *ROS1*, *ARID1A*, *PBRM1*, *BRCA1*, and *BAP1* (8–16). FGFR kinase inhibitors have demonstrated anti-tumor activity in ICC patients harboring activating FGFR2 gene fusions (17–19). However, no effective therapeutic strategies have currently changed the standard of care of ICC patients harboring different types of alterations.

Here, we describe the case of a chemorefractory patient with ICC harboring BAP1 mutation and RAD21 amplification. The patient was successfully treated with the PARP inhibitor olaparib.

## Case Presentation

**Figure 1:**
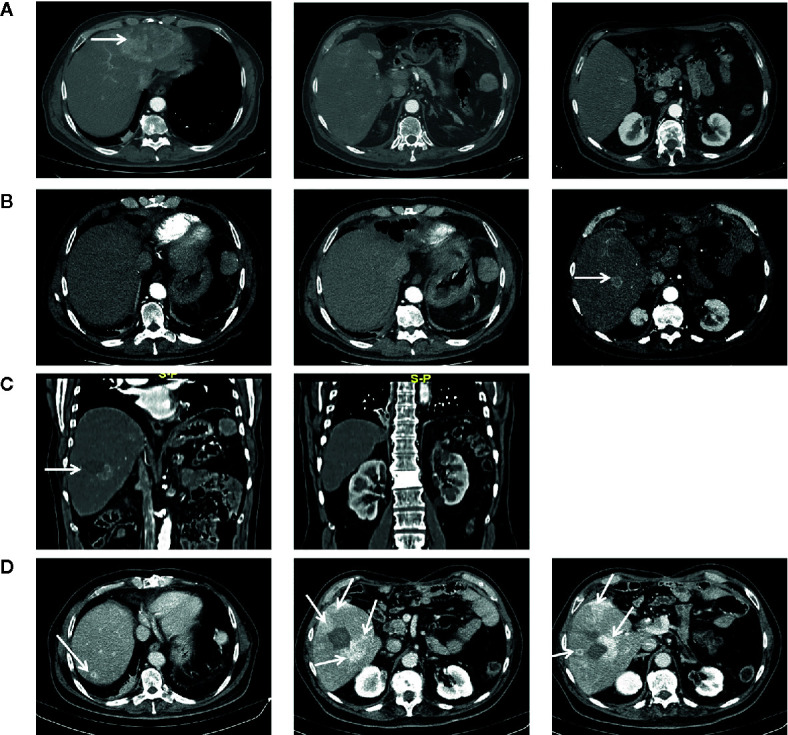
Chest CT-scan performed at diagnosis in March 2017 **(A)**, in October 2017 following first relapse **(B)**, in February 2018 at tumor progression following first percutaneous thermal ablation **(C)**, in May 2018 at tumor progression following second percutaneous thermal ablation and before starting chemotherapy with cisplatin and gemcitabine **(D)**. Arrows indicate tumor lesion.

**Figure 2:**
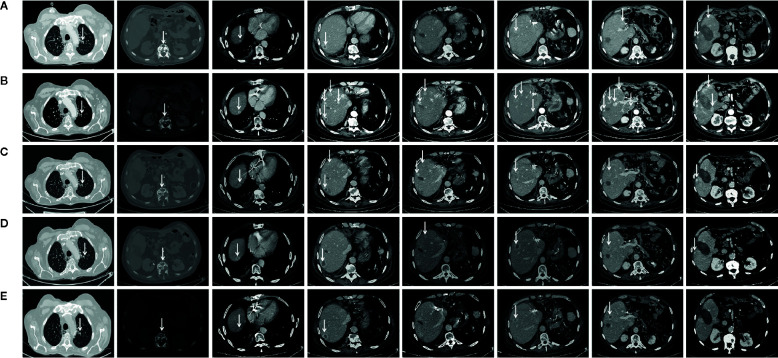
Chest CT-scan performed at diagnosis in February 2019 at tumor progression following chemotherapy with cisplatin and gemcitabine and a third percutaneous thermal ablation and before to start treatment with FOLFIRI **(A)**, in June 2019 at tumor progression following six cycles of FOLFIRI administration and before to start treatment with olaparib **(B)**, in September 2019 following three cycles of olaparib **(C)**, in November 2019 following six cycles of olaparib **(D)**, and in February 2020 following 11 cycles of olaparib **(E)**. Arrows indicate tumor lesion.

**Figure 3:**
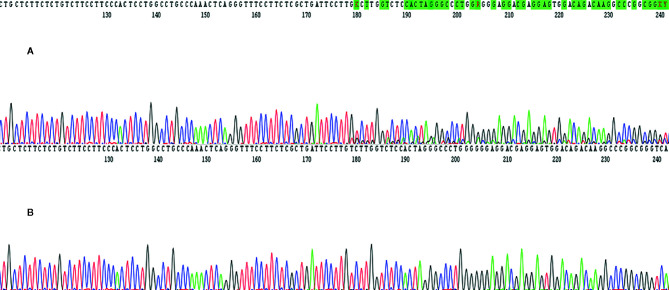
The figure shows BAP1 molecular analysis performed on tumor tissue samples and buffy coat by using sanger sequencing platform. In details, c. 581-17_585del22 mutation was found only in tumor tissue specimen **(A)** while nucleic acids extracted from buffy coat did not harbor this mutation **(B)**.

In March 2017, a 56-year-old Caucasian male was admitted to San Giovanni di Dio and Ruggi D’Aragona University Hospital for mild abdominal pain and nausea. The patient’s past medical history included i) Hodgkin’s lymphoma of the spleen in 1987, treated with splenectomy and radiotherapy; ii) myocardial infarction in 2006, treated with coronary angioplasty; and iii) myocardial infarction in 2012, treated with multiple coronary artery bypass grafting. He was also a former-smoker. Patient did not present with any ICC risk factors including biliary lithiasis, alcoholic liver disease, chronic hepatitis B or C infections, or primary sclerosing cholangitis. His family history was negative for any inherited-familial cancers. Abdominal ultrasound and computed tomography (CT) scan revealed a 10 cm intrahepatic lesion in the left lobe of the liver, as well as stable right basal lung thickening (**Figure 1A**). The latter was already described in a previous chest CT scan. Ultrasound guided biopsy of the liver mass demonstrated ICC (CK7+, CK19+, HepPar1-, AFP-). In April 2017, the patient underwent a left hepatectomy and sub-total gastrectomy and cholecystectomy. Histological examination demonstrated a Stage II ICC with vascular invasion [TNM staging, American Joint Committee on Cancer (AJCC) 8^th^ edition]. Post operatively he was seen by the multidisciplinary team. Genomic analysis of *NRAS*, *KRAS* and *BRAF V600* by polymerase chain reaction (PCR) sequencing, as well as immunohistochemical (IHC) staining for detection of HER2 amplification were performed on ICC tumor tissue. Both analyses did not show any type of alteration (**Supplementary Table 1**). Further genomic testing of *EGFR* was performed by sanger sequencing, but no alterations were found in exons 18, 19, 20, and 21 (**Supplementary Table 1**). In October 2017, a whole body CT scan demonstrated a 2.0 cm local recurrence in segment V of the liver (**Figure 1B**). Patient received a percutaneous thermal ablation (PTA) of the lesion. In February 2018, a whole body CT scan demonstrated a new 3.6 cm local recurrence in segment V of the liver, close to the previously treated lesion (**Figure 1C**) for which patient received a new PTA. In May 2018, a whole body CT scan demonstrated a new local recurrence in segment V of liver and multiple lesions in segment VII and VIII (**Figure 1D**). He then started a chemotherapeutic regimen with cisplatin (25 mg/m^2^) followed by gemcitabine (1,000 mg/m^2^), each administered on days 1 and 8 every 3 weeks. Due to his poor prognosis, patient requested additional testing of the ICC specimen. An IHC analysis of ROS1 rearrangements and NTRK fusions did not demonstrate any alterations (**Supplementary Table 2**). A Short Tandem Repeat (STR) analysis by PCR of *BAT25*, *BAT26*, *D2S123*, *D5S346*, *D17S250*, *NR-21*, and *MONO-27* showed a Microsatellite Stable (MSS) tumor profile. Lastly an IHC analysis of MSH2, MSH6, PMS2, and MLH1 demonstrated no alterations of the mismatch repair system (**Supplementary Table 2**). Following six cycles of cisplatin and gemcitabine, in September 2018, a whole-body CT scan demonstrated a stable disease (according to RECIST criteria v 1.1). The patient received an additional PTA of the lesions in segments V, VII, and VIII of the liver. In February 2019, the CT scan demonstrated progression of disease (PD) (according to RECIST criteria v 1.1) due to the development of multiple small lesions localized at the hepatic dome and around the area of previous PTA, long with a large bone metastasis to the 12^th^ vertebral body and a left upper lobe pulmonary nodule (**Figure 2A**). Based on the availability of additional formalin fixed tumor tissue obtained from a novel tumor biopsy, three different NGS platform studies were requested by the patient: Oncomine Comprehensive Assay (implemented at Istituto Tumori Milano, Milan, Italy) (**Table 1**), Oncofocus test [Oncologica^®^ UK ltd (Cambridge, UK)] (**Table 2**) and Foundation One CDx [Foundation Medicine (Cambridge, MA)] (**Table 3**). Both the Oncomine Comprehensive Assay and the Oncofocus test did not detect any alterations of analyzed genes. In contrast the Foundation One CDx demonstrated the presence of a deletion in *BAP1* (splice site c.581-17_585del22) and amplification of *RAD21.* Analysis of *BAP1* by sanger sequencing on primary ICC tumor tissue confirmed the presence of BAP1 (splice site 581-17_585del22) alteration (**Figure 3**). In contrast no alterations were identified in *BAP1* from nucleic acids extracted from buffy coat (**Figure 3**). Because of the involvement of RAD21 in the DNA repair pathway, the interaction of BAP1 with BRCA1 and the enhanced sensitivity to PARP inhibitor administration in presence of alterations in the BRCA1-mediated DNA repair pathway, it was decided first to treat the patient with FOLFIRI every 2 weeks [irinotecan 180 mg/m^2^, folinic acid 400 mg/m^2^, 5-fluorouracil (5-FU) 400 mg/m^2^ intravenous infusion bolus, then 5-FU 2400 mg/m^2^ intravenous infusion over 46 h] and then to start a PARP inhibitor. FOLFIRI is a conventional second-line chemotherapy regimen for ICC. In addition, irinotecan is a DNA-damaging agent. Following six cycles of FOLFIRI, in June 2019, a whole-body CT scan demonstrated PD (**Figure 2B**). A third-line therapy of off-label use with the PARP inhibitor olaparib at 800 mg/die and palliative radiotherapy (10 Gy) on the vertebral lesion was begun. In September 2019, a whole-body CT scan demonstrated a partial response (PR) (**Figure 2C**). The latter was confirmed on successive restaging scans in November 2019 (**Figure 2D**) and February 2020 (**Figure 2E**). Following 11 cycles of olaparib, the progression free survival has been 11.0 months. Currently, the patient has an overall survival of 37.2 months from the time of diagnosis of his ICC and has continued treatment with olaparib. He is in good health conditions and no treatment-related adverse events have been reported.

**Table 1:** Oncomine Comprehensive Assay.

March 1^st^, 2019								
NGS: Hot spot Cancer Panel with PGM (Personal Genome Machine) Ion Torrent technology [Thermo Fisher Scientific Life Technologies (Waltham, MA)]								
ABL1	AKT1	ALK	APC	ATM	BRAF	CDH1	CDKN2A	CSF1R
CTNNB1	EGFR	ERBB2	ERBB4	EZH2	FBXW7	FGFR1	FGFR2	FGFR3
FLT3	GNA11	GNAQ	GNAS	HNF1A	HRAS	IDH1	IDH2	JAK2
JAK3	KDR (VEGFR2)	KIT	KRAS	MET	MLH1	MPL	NOTCH1	NPM1
NRAS	PDGFRA	PIK3CA	PTEN	PTPN11	RB1	RET	SMAD4	SMARCB1
SMO	SRC	STK11	TP53	VHL				
Results: No hot spot mutations detected.								

**Table 2:** Oncofocus test.

March 2^nd^, 2019														
NGS: Oncofocus test (Oncologica^®^ UK ltd (Cambridge, UK)														
A2M	ABCB5	ACACA	ACADM	ACBD5	ACTG2	ADAM32	ADAMTS16	AES	AFAP1	AFF3	AGAP3	AGBL4	AGGF1	AGK
AGTRAP	AHCYL1	AKAP12	AKAP13	AKAP9	AKT1	AKT2	AKT3	ALK	AP3B1	AR	ARAF	ARHGEF2	ARID1A	ARMC10
ARMT1	ASIC2	ATAD2	ATAD5	ATF7IP	ATG7	ATIC	ATM	ATP1B1	ATR	ATRNL1	ATRX	AXL	B4GALT1	BAG4
BAIAP2L1	BAP1	BBS9	BCAM	BCAN	BCL2L11	BCR	BEND5	BICC1	BICD2	BIN2	BIRC6	BRAF	BRCA1	BRCA2
BRD3	BRD4	BTAF1	BTBD1	BTF3L4	BTK	C11orf95	C7orf73	C8ORF34	C9orf153	CAD	CAND1	CAPRIN1	CAPZA2	CARS
CASP7	CBL	CCAR2	CCDC170	CCDC6	CCDC88A	CCDC91	CCND1	CCND2	CCND3	CCNE1	CCNY	CD44	CD74	CDC27
CDK12	CDK2	CDK4	CDK5RAP2	CDK6	CDKN1B	CDKN2A	CDKN2B	CEL	CEP85L	CEP89	CHD9	CHEK1	CHEK2	CHTOP
CIC	CIITA	CIT	CLCN6	CLIP1	CLIP2	CLIP4	CLTC	CNTLN	CNTRL	COL14A1	COX5A	CPSF6	CREB3L2	CREB5
CREBBP	CSF1R	CTNNB1	CUL1	CUX1	DAB2	DAB2IP	DCTN1	DDR2	DIP2C	DNAJB1	DTD1	DYM	DYNC1I2	DYNC2H1
EBF1	EGFR	EIF3E	ELAVL3	EML4	EPHB2	EPS15	ERBB2	ERBB3	ERBB4	ERC1	ERCC2	ERG	ERLIN2	ERP44
ERVK3_1	ESR1	ESRP1	ETV1	ETV4	ETV5	ETV6	EZH2	EZR	FAM114A2	FAM131B	FAM76A	FANCA	FANCD2	FANCI
FA1	FBXO28	FBXW7	FCHSD1	FGF3	FGFR1	FGFR19	FGFR1OP	FGFR1OP2	FGFR2	FGFR3	FGFR4	FGR	FP1L1	FKBP15
FLT3	FN1	FNDC3B	FOXL2	FOXP1	FXR1	FYCO1	GABBR2	GATA2	GATM	GFPT1	GHR	GIT2	GLIS3	GNA11
GNAI1	GNAQ	GNAS	GNS	GOLGA4	GOLGA5	GOLGB1	GOPC	GRB7	GRHL2	GTF2I	GTF2IRD1	GTF3C2	H3F3A	HACL1
HERPUD1	HIP1	HIST1H3B	HLA_A	HMGA2	NHNF1A	HOMER1	HOOK3	HRAS	IDH1	IDH2	IGF1R	IRF2BP2	JAK1	JAK2
JAK3	JAKMIP1	KANK1	KANK2	KCNQ5	KCTD1	KCTD7	KDELR2	KDM7A	KDR	KIAA1468	KIAA1549	KIAA1598	KIF5B	KIT
KLC1	KLHL7	KNSTRN	KRAS	KTN1	LMNA	LRIG3	LRRFIP1	LSM12	LSM14A	LYN	MACF1	MAD1L1	MAGOH	MAP2K1
MAP2K2	MAP2K4	MAPK1	MAX	MBIP	MCFD2	MDM2	MDM4	MED12	MEMO1	MET	MGEA5	MIR143HG	MKRN1	MLH1
MPRIP	MRE11A	MRPL24	MRPS33	MSH2	MSH6	MSN	MTFHD1L	MTMR12	MTOR	MYB	MYBL1	MYC	MYCL	MYCN
MYD88	MYH13	MYH9	MYO18A	MYO5A	MYRIP	MZT1	NACC2	NAV1	NBN	NCOA1	NCOA4	NCOR2	NDE1	NF1
NF2	NFASC	NFIB	NFKB2	NIN	NOL4	NOTCH1	NOTCH2	NOTCH3	NOTCH4	NPC2	NPM1	NRAS	NRG1	NSD1
NTM	NTRK1	NTRK2	NTRK3	NUB1	NUDCD3	NUP214	NUTM1	OFD1	OPHN1	OXR1	PALB2	PAPD7	PAPSS1	PARK2
PAX5	PAX8	PCDHGA1	PCM1	PCNX	PDE10A	PDE4DIP	PDE7A	PDGFRA	PDGFRB	PDHX	PDP1	PDZRN3	PHEB	PIK3CA
PIK3CB	PIK3R1	PLAG1	PLIN3	PMS2	POLE	POLH	PPARG	PPFIBP1	PPHLN1	PPL	PPM1G	PPP2R1A	PPP4R3B	PRKACA
PRKACB	PRKAR1A	PRKG2	PSMD11	PSPH	PTCH1	PTEN	PTPN11	PTPN3	PTPRK	PTPRZ1	PWWP2A	QKI	RABEP1	RABGAP1L
RAC1	RAD18	RAD50	RAD51	RAD51B	RAD51C	RAD51D	RAF1	RANBP2	RB1	RBMS3	RBPMS	RELA	RET	RHOA
RICTOR	RNF11	RNF130	RNF213	RNF43	ROS1	RP2	RSPO2	RSPO3	RUFY2-	SART3	SCAF11	SDC4	SDCCAG3	SEC16A
SEC31A	SEC61G	SETD2	SF3B1	SHROOM4	SHTN1	SLC12A7	SLC26A4	SLC34A2	SLC3A2	SLC45A3	SLMAP	SLX4	SMAD4	SMARCA4
SMARCB1	SMOP	SND1	SNHG7	SNX19	SOX6	SPAG9	SPECC1	SPECC1L	SPOP	SPTBN1	SQSTM1	SRC	SRGAP3	SSBP2
STAT3	STK11	STK32B	STRN	STRN3	SUGCT	TACC1	TACC3	TANK	TAX1BP1	TBL1XR1	TENM4	TERF2	TERT	TPM1
TFG	TMEM106B	TMEM178B	TMPRSS2	TNIP1	TNKS2	TOP1	TP53	TP53BP1	TPM3	TPM4	TPR	TRAF1	TRAK1	TRIM24
TRIM27	TRIM33	TRIM4	TRIO	TRIP11	TRMT61B	TSC1	TSC2	TSEN2	TTLL7	TXLNA	TYK2	U2AF1	UBE2L3	UBN2
USP10	VAMP2	VCL	VOPP1	WASF2	WDR48	WHSC1L1	WIPF2	XPO1	YAP1	YTHDF3	YWHAE	ZC3HAV1	ZCCHC8	ZEB2
ZKSCAN1	ZKSCAN5	ZMYM2	ZMYND8	ZNF226	ZNF703	ZSCAN30								
Results: - Mutations: No actionable variant detected - Copy Number Variations: No actionable variant detected - Fusion Genes: No actionable variant detected														

**Table 3:** Foundation One CDx.

March 7^nd^, 2019												
DNA GENE LIST: ENTIRE CODING SEQUENCE FOR THE DETECTION OF BASE SUBSTITUTIONS, INSERTION/DELETIONS, AND COPY NUMBER ALTERATIONS Foundation One CDx [Foundation Medicine (Cambridge, MA)]												
ABL1	ACVR1B	AKT1	AKT2	AKT3	ALK	ALOX12B	AMER1 (FAM123B)		APC	AR	ARAF	ARFRP1
ARID1A	ASXL1	ATM	ATR	ATRX	AURKA	AURKB	AXIN1		AXL	BAP1	BARD1	BCL2
BCL2L1	BCL2L2	BCL6	BCOR	BCORL1	BRAF	BRCA1	BRCA2		BRD4	BRIP1	BTG1	BTG2
BTK	C11orf30 (EMSY)	C17orf39 (GID34)	CALR	CARD11	CASP8	CBFB	CBL		CCND1	CCND2	CCND3	CCNE1
CD22	CD274 (PD-L1)	CD70	CD79A	CD79B	CDC73	CDH1	CDK12		CDK4	CDK6	CDK8	CDKN1A
CDKN1B	CDKN2A	CDKN2B	CDKN2C	CEBPA	CHEK1	CHEK2	CIC		CREBBP	CRKL	CSF1R	CSF3R
CTCF	CTNNA1	CTNNB1	CUL3	CUL4A	CXCR4	CYP17A1	DAXX		DDR1	DDR2	DIS3	DNMT3A
DOT1L	EED	EGFR	EP300	EPHA3	EPHB1	EPHB4	ERBB2		ERBB3	ERBB4	ERCC4	ERG
ERRFI1	ESR1	EZH2	FAM46C	FANCA	FANCC	FANCG	FANCL		FAS	FBXW7	FGF10	FGF12
FGF14	FGF19	FGF23	FGF3	FGF4	FGF6	FGFR1	FGFR2		FGFR3	FGF4	FH	FLCN
FLT1	FLT3	FOXL2	FUBP1	GABRA6	GATA3	GATA4	GATA6		GNA11	GNA13	GNAQ	GNAS
GRM3	GSK3B	H3F3A	HDAC1	HGF	HNF1A	HRAS	HSD3B1		ID3	IDH1	IDH2	IGF1R
IKBKE	IKZF1	INPP4B	IRF2	IRF4	IRS2	JAK1	JAK2		JAK3	JUN	KDM5A	KDM5C
KDM6A	KDR	KEAP1	KEL	KIT	KLHL6	KMT2A (MLL)	KMT2D (MLL2)		KRAS	LTK	LYN	MAF
MAP2K1 (MEK1)	MAP2K2 (MEK2)	MAP2K4	MAP3K1	MAP3K13	MAPK1	MCL1	MDM2		MDM4	MED12	MEF2B	MEN1
MEERTK	MET	MITF	MKNK1	MLH1	MPL	MRE11A	MSH2		MSH3	NBN	NF1	NF2
NFE2L2	NFKBIA	NKX2-1	NOTCH1	NOTCH2	NOTCH3	NPM1	NRAS		NSD3 (WHSC1L1)	NT5C2	NTRK1	NTK2
NTRK3	P2RY8	PALB2	PARK2	PARP1	PARP2	PARP3	PAX5		PBRM1	PRKAR1A	PRKCI	PTCH1
PTEN	PTPN11	PTPRO	QKI	RAC1	RAD21	RAD51	RAD51B		RAD51C	RAD51D	RAD52	RAD54L
RAF1	RARA	RB1	RBM10	REL	RET	SF3B1	SGK1		SMAD2	SMAD4	SMARCA4	SMARCB1
SMO	SNCAIP	SOCS1	SYK	TBX3	TEK	TET2	TGFBR2		TIPARP	TNFAIP3	TNFRSF14	TP53
TSC1	TSC2	TYRO3	U2AF1	VEGFA	VHL	WHSC1	WT1		XPO1			
**DNA GENE LIST: FOR THE DETECTION OF SELECT REARRANGEMENTS**												
ALK	BCL2	BCR	BRAF	BRCA1	BRCA2	CD74	EGFR	ETV4		ETV5	ETV6	EWSR1
EZR	FGFR1	FGFR2	FGFR3	KIT	KMT2A (MLL)	MSH2	MYB	MYC		NOTCH2	NTRK1	NTRK2
NUTM1	PGFRA	RAF1	RARA	RET	ROS1	RSPO2	SDC4	SLC34A2		TERC	TERT	TMPRSS2
**Results:** - **BAP1: Splice site 581-17_585del22** - **RAD21: amplification**												

## Discussion and Conclusions

Novel effective therapies are urgently needed for metastatic ICC patients. The current clinical case has provided for the first-time evidence that ICC patients carrying a BAP1 deletion and RAD21 amplification might benefit from a PARP inhibitor treatment. *BAP1* is a tumor suppressor gene which modulates several pathways including cell death, cell differentiation, DNA damage response and gluconeogenesis (20–28). In mediating DNA damage response, BAP1 interacts with BRCA1 (20, 21). BRCA1 plays a key role in the DNA repair mechanism as well as in cell cycle regulation (29). Germline heterozygous mutations in *BAP1* cause an autosomal dominant condition known as BAP1-cancer syndrome which confers a high susceptibility to the development of several malignancies including mesothelioma, uveal melanoma, renal, cholangio and breast carcinomas (30–38). In the clinical case we have described, we identified a novel mutation in *BAP1* (c.581-17_585del22). The variant was somatic and not detected in the germline. We have examined several databases (Cosmic, GenBank, ClinVar) and c.581-17_585del22 mutation was not identified. Some literature data reported a similar deletion of *BAP1* with a pathogenic value (39, 40). Somatic mutations in *BAP1* are reported to drive carcinogenesis in mesothelioma, lung adenocarcinoma and melanoma (30, 32, 34, 41). BAP1 mutations occur in 10–32% of ICC cases (10, 14, 30, 42–49). As a tumor suppressor gene, BAP1 seems to follow a classic two-hit model (Knudson model) in which probably the first hit involves loss of heterozygosity (LOH) induced by 3p21 deletion. The latter occurs in almost 50–75% of ICCs (36). A subsequent mutation occurring in the remaining allele might lead to impairment of protein function and/or homeostasis (36). Protein function impairment by c.581-17_585del22 is most likely to reflect a deletion in the 3’-splice site of *BAP1*. Previously a c.581(-5)_c.590delACTAGGGCCCTGGGG mutation has been reported causing a premature truncation of BAP1 (50). This type of alterations that disrupt the nuclear localizations signal (aminoacids 717-722) of *BAP1* are predicted to be inactivating (14, 51).

As BAP1 interacts with BRCA1, several lines of evidence indicate that alterations in the BRCA-mediated DNA repair pathway confers sensitivity to PARP inhibitor administration (52). PARP inhibitors act through synthetic lethality, whereby genetic DNA repair defects are enhanced by drug-induced defects in a compensatory pathway (53). Carriers of heterozygous BRCA1/2 mutations are sensitive to PARP inhibitor treatment as they lose the wild-type allele during tumorigenesis and thereby become deficient of the homologous recombination (HR) pathway of double-strand break DNA repair by BRCA1/2-null status. Four PARP inhibitors, olaparib, rucaparib, niraparib, and talazoparib, have been approved by the U.S. Food and Drug Administration (FDA) and by the European Medicines Agency (EMA). In 2014, olaparib was approved as maintenance therapy for platinum-sensitive advanced ovarian cancer with germline mutations in BRCA1/2. In 2016, rucaparib was approved for advanced ovarian cancer with both germline and somatic BRCA1/2 mutations. In 2017 and 2018, olaparib, rucaparib, and niraparib were approved for the maintenance treatment of recurrent, epithelial ovarian, fallopian tube, or primary peritoneal cancer irrespective of the BRCA status. Last, in 2018, olaparib and talazoparib were approved for HER2-negative locally advanced or metastatic breast cancer with germline BRCA1/2 mutations. Besides in ovarian and breast cancer, PARP inhibitor efficacy has also been demonstrated in other types of cancer including prostate and pancreatic cancer, and small cell lung carcinoma, irrespective of the BRCA status (54–61). It has become clear that any form of HR deficiency in tumors that phenocopies BRCA1/2 mutations, often referred to as BRCAness, may sensitize cells to PARP inhibitors (62). Indeed mutations in DNA damage response genes such as *ATM*, *PRKDC*, *ATR*, *RPA1*, *DSS1*, *NBN*, *RAD51*, *RAD54*, *CHEK1*, *CHEK2*, FANC genes, *ERCC1*, *POLB*, *FEN1*, and *CDK12* have shown synthetic lethality in combination with PARP inhibitors (63–67).

BAP1 is a HR DNA repair component and its loss sensitizes cancer cells to DNA repair defects (28). Currently, further investigations are needed to establish the real efficacy of PARP inhibitor on BAP1 mutated cancer cells. Some studies on various types of BAP1 mutated cancer cell lines demonstrated the potential efficacy of PARP inhibitors (68–70). A synergistic effect of PARP inhibitor and gemcitabine is described in BAP1 deficient cholangiocarcinoma cell lines (71). As a result, PARP inhibitors are currently under investigation alone or in combination with other therapies in cancer patients harboring a BAP1 mutant tumor including ICC (ClinicalTrials.gov Identifier: NCT03207347, NCT03786796, NCT03531840, and NCT03375307).

In the current clinical case, we have shown that PARP inhibitor administration can be potentially effective in BAP1 mutated ICC. Chemotherapeutic agents, such as platinum compounds which induce double-strand DNA breaks, are usually utilized prior to PARP inhibition in order to enhance DNA damage and induce PARP inhibition-mediated cell death (72). In addition PARP inhibitors are currently administered after obtaining a disease control with platinum compounds (73, 74). In the present clinical case, the PARP inhibitor olaparib was effective in controlling tumor progression, even though the patient did not benefit from FOLFIRI administration, a combination of 5-FU and topoisomerase I inhibitor irinotecan. Irinotecan exerts its anticancer effects through induction of single- and double-strand DNA breaks. 5-FU is an antimetabolite drug that exerts its anticancer effects through inhibition of DNA synthesis by inhibition of thymidylate synthase and incorporation of its metabolites into RNA and DNA. One could speculate that efficacy to PARP inhibitor was not enhanced by FOLFIRI administration, but rather by the previous administration of cisplatin. Additional studies are needed to define the timing and schedule of DNA damaging agents for PARP inhibitor enhancement in BAP1 deficient tumors.

In addition to BAP1 mutations, many other molecular alterations have been described in ICC such as *KRAS*, *BRAF*, *IDH1, IDH2*, *EGFR*, *FGFR2*, *ROS1*, *ARID1A*, *PBRM1*, and *BRCA1* (8–16). These types of alterations are frequently mutually exclusive (8–16). In the current clinical case, BAP1 mutation is not associated with KRAS, BRAF, IDH1, IDH2, EGFR, FGFR2, ROS1, ARID1A, PBRM1, and BRCA1 alterations but with a RAD21 amplification. Further studies are needed to validate this type of association. *RAD21* is a gene involved in the repair of DNA double-strand breaks, as well as in chromatid cohesion during mitosis (75, 76). Amplification of *RAD21* is described in approximately 1.23% of cases reported in the AACR Project Genomics Evidence Neoplasia Information Exchange (AACR Project GENIE), including invasive breast carcinoma, prostate adenocarcinoma, lung adenocarcinoma and colon adenocarcinoma having the greatest prevalence (77). However, no prior data exists regarding RAD21 amplification in ICC. Whether RAD21 amplification might enhance the activity of a PARP inhibitor in BAP1 mutant ICC should be further investigated.

Both BAP1 and RAD21 alterations were detected by utilizing NGS analysis. Patient’s tumor tissue underwent analysis by several precision oncology testing methods to identify potentially oncogenic alterations. However, most of the tests performed did not detect any alterations. By comparing the results from the two most extensive tumor genomic profiles *BAP1* was analyzed in both: the Foudation One CDx and Oncofocus test. However only the Foudation One CDx test was able to detect BAP1 and RAD21 alterations. These findings are likely to reflect the different methods utilized to detect potentially oncogenic alterations, the regions of the genes included in the analysis, the potential tumor heterogeneity especially with a low allele frequency of the variants and the percentage of tumor cells in the sample tested. Since there is no targeted regions for BAP1 it is unlikely that different NGS platforms only test selected exons. In our case the novel mutation c.581-17_585del22 of BAP1 was localized on exon 8 of *BAP1*, at the boundary of intron 7. Most of the NGS platforms include 20-25bp in the vicinity of exons. However the Oncofocus^®^ Test did not detect the c.581-17_585del22 alteration of BAP1 alteration most likely because this region of the gene was not included in the analysis. In contrast, the Foundation One CDx platform included in the analysis the full exonic region of *BAP1* besides including also *RAD21* in the analysis. Foundation One CDx report contains information only about the genomic findings without allele frequency values. As limit of detection range at non-homopolymer context (insertion up to 42 bp and deletion up to 276 bp) is 6–10%, we can assume that the BAP1 c.581-17_585del mutated allele was present with a higher variant fraction in the metastatic tumor tissue analyzed. In addition, direct sequencing has a reported limit of detection of approximately 20% mutant alleles. In our case BAP1 sanger sequencing on primary ICC tumor tissue showed the unbalanced presence of the mutated allele, even if it is not possible to have a quantitative value, as with NGS or digital PCR, we can hypothesize an allele frequency close to the limit of detection. Therefore, we can assume that BAP1 c.581-17_585del mutated allele occurred with a high allele frequency, early in ICC oncogenesis.

In conclusion, genomic characterization of ICC tumors by NGS analysis can identify potential targetable oncogenic alterations in ICC, providing the possibility to improve patient survival. Specifically, BAP1 deletion and RAD21 amplification were identified and effectively targeted by PARP inhibitor administration. These results warrant further studies to define the role of PARP inhibitor in ICC harboring BAP1 and RAD21 alterations.

## Data Availability Statement

The original contributions presented in the study are included in the article/**Supplementary Material**. Further inquiries can be directed to the corresponding author. 

## Ethics Statement

Written informed consent was obtained from the patient for publication of this case report and any accompanying images.

## Author Contributions

Conception and design: FSa, SP, and UM. Acquisition of data: LL, VT, and FSa. Analysis and interpretation of data: FSa, AF, VC, FSc, and UM. Writing, review, and/or revision of the manuscript: FSa, LL, and CF. Administrative, technical, or material support (i.e., reporting or organizing data, constructing databases): LL, and VT. Study supervision: SP. Other (contributed clinical and pathological material; discussed results and implications of findings): SP, GT, and CF. All authors contributed to the article and approved the submitted version.

## Funding

The work was supported by Ministero dell’Università e della Ricerca (Progetti di Rilevante Interesse Nazionale (PRIN), 2017, CODICE 2017PHRC8X_003) (to SP).

## Conflict of Interest

UM reports personal fees (as speaker bureau or advisor) from Boehringer Ingelheim, AstraZeneca, Roche, MSD, Amgen and Merck, unrelated to the current work.

The remaining authors declare that the research was conducted in the absence of any commercial or financial relationships that could be construed as a potential conflict of interest.

## Abbreviations

AFP, alpha fetoprotein; AJCC, American Joint Committee on Cancer; ARID1A, AT-rich interactive domain-containing protein 1A; ATM, ataxia-telangiectasia mutated; ATR, ataxia telangiectasia and Rad3-related protein; BAP1, BRCA1 associated protein 1; BRAF, v-raf murine sarcoma viral oncogene homolog B1; BRCA1, Breast cancer type 1 susceptibility protein; CCA, Cholangiocarcinoma; CDK12, Cyclin Dependent Kinase 12; CHEK1-CHEK2, Checkpoint kinase 1-2; CK7, Cytokeratin 7; CK19, Cytokeratin 19; CT, computed tomography; EGFR, Epidermal Growth Factor Receptor; EMA, European Medicines Agency; ERCC1, excision repair cross-complementation group 1; FANC, Fanconi anaemia complementation group; FDA, Food and Drug Administration; FEN1, Flap endonuclease 1; FFPE, formalin fixed paraffin embedded; FGFR2, Fibroblast Growth Factor Receptor 2; 5-FU, 5-fluorouracil; HepPar1, Hepatocyte Paraffin 1; HER2, Human epidermal growth factor receptor 2; HR, homologous recombination; ICC, Intrahepatic cholangiocarcinoma; IDH1/IDH2, Isocitrate Dehydrogenase 1/2; IHC, immunohistochemical; KRAS, Kirsten ras oncogene homolog; LOH, loss of heterozygosity; MSS, Microsatellite Stable; NBN, Nibrin; NGS, next-generation sequencing; NRAS, neuroblastoma; RAS, viral oncogene homolog; RPA1, Replication protein A 70 kDa DNA-binding subunit; NTRK, neurotrophic receptor tyrosine kinase 1; PARP, poly ADP ribose polymerase; PCR, polymerase chain reaction; PR, partial response; PBRM1, Polybromo 1; PCR, polymerase chain reaction; PD, progression of disease; POLB, DNA Polymerase Beta; PRKDC, Protein Kinase; DNA-Activated, Catalytic Subunit; PTA, percutaneous thermal ablation; RAD51-RAD54, radiation-repair genes 51–54; RECIST, Response evaluation criteria in solid tumors; ROS1, ROS proto-oncogene 1receptor tyrosine kinase; STR, Short Tandem Repeat.
